# Evaluation of non-motor symptoms and their impact on quality of life in patients with Parkinson’s disease, Isfahan, Iran

**Published:** 2017-07-06

**Authors:** Mehri Salari, Ahmad Chitsaz, Masoud Etemadifar, Mohammad Reza Najafi, Omid Mirmosayyeb, Maryam Bemanalizadeh, Fatemeh Panahi, Hosna Mirzajani

**Affiliations:** 1 Department of Neurology, School of Medicine, Isfahan University of Medical Sciences, Isfahan, Iran; 2 Isfahan Neurosciences Research Center, Alzahra Research Institute, Isfahan University of Medical Sciences, Isfahan, Iran; 3 Student Research Committee, Isfahan University of Medical Sciences, Isfahan, Iran

**Keywords:** Non-Motor Symptoms, Parkinson Disease, Quality of Life

## Abstract

**Background:** Parkinson’s disease (PD) is diagnosed on the basis of motor symptoms, but non-motor symptoms (NMS) have high prevalence in PD and often antecede motor symptoms for years and cause severe disability. This study was conducted to determine the prevalence of NMS in patients with PD.

**Methods:** This cross-sectional study was performed in Isfahan, Iran, on patients with PD. The prevalence of NMS was evaluated by the NMS questionnaire, the NMS scale, and Parkinson's disease questionnaire-39 (PDQ-39). The Mini-Mental Status Examination (MMSE) was used for assessing cognition.

**Results:** A total of 81 patients, including 60 men and 21 women, were recruited for this study. The prevalence of NMS was 100%, and the most commonly reported symptom was fatigue (87.7%); there was a strong correlation between NMS and the quality of life (QOL) of patients with PD (P < 0.001).

**Conclusion:** This study showed that NMS are highly prevalent in the PD population and adversely affect QOL in these patients. Early diagnosis and treatment can improve QOL and can help in disability management of patients with PD.

## Introduction

Although non-motor symptoms (NMS) are common among patients with Parkinson’s disease (PD), they are often not well-known in clinical practice.^1^ While PD is diagnosed on the basis of motor symptoms, comprising slowness of movement and complications with balance, it is known that NMS are highly prevalent and often amenable to therapy.^2^ In fact, studies have demonstrated that NMS of PD, such as sleep disturbances, anxiety, and depression, are more disabling than motor symptoms of PD, deteriorating the quality of life (QOL). NMS are also the most common reason for admission to institutional care.^3^

The pathophysiology of NMS is still poorly understood, and dysfunction of both dopaminergic and non-dopaminergic systems seem to be involved.^3^ Although the non-motor features of PD are common, these symptoms are not frequently distinguished in clinical practice. It has been found that approximately half of the NMS of PD are not recognized even by neurologists, causing interruptions in treatment and insufficient management. There are precise validated tools available for NMS assessment, such as the NMS questionnaire (NMSQ) and NMS scale (NMSS).^4^

Recognizing the most prevalent NMS in PD and clarifying their clinical features would help in the diagnosis of PD prior to the presence of motor symptoms.^5^ To describe the range and prevalence of NMS in patients diagnosed with PD, we enrolled participants in a cross-sectional study by the unified PD rating scale (UPDRS), NMSQ, NMSS, and Parkinson’s disease questionnaire-39 (PDQ-39).

Moreover, the Mini-Mental Status Examination (MMSE) and a comprehensive medical history of all patients were taken to detect the correlations between NMS and PD.

## Materials and Methods

This observational cross-sectional study was prospectively performed on patients with idiopathic PD (IPD) diagnosed on the basis of the UK brain bank criteria; the patients were recruited by referral from a PD clinic in the Al-Zahra hospital, Isfahan, Iran. Written informed consent was obtained from the patients for the time period of June 2014–June 2015. The Ethical Committee of the Isfahan University of Medical Sciences approved the study (approval code: 294011).

All patients diagnosed with IPD who agreed to participate underwent medical evaluation by a neurologist specialized in movement disorders, and who had a certificate from a MDS-UPDRS training program. Medical students completed the questionnaires by interviewing patients or their care givers. Patients with neurological or systemic diseases that could affect NMS and QOL (disability due to cerebrovascular disease, advanced diabetes mellitus, renal failure, heart failure, hepatic failure, malignancy, severe anemia, pain syndrome) and patients with severe cognitive impairment (MMSE < 19) that could cause unreliable information were excluded from the study.^6^

Demographic data, disease history, and social information were collected by a checklist, the modified Hoehn and Yahr staging.^7^ The Unified Parkinson’s Disease Rating Scale-part III (UPDRS-III)^8^ was used for assessment of motor symptom severity, and NMS were evaluated by the NMSQ,^9^ NMSS,^10^ and UPDRS I-II. For QOL, PDQ-39^11^ was used. UPDRS-IV was used to assess motor complications, and finally, cognitive abilities were investigated with the MMSE.^12^

***Questionnaire:*** 1- UPDRS, which adjusted according to the MDS-UPDRS revision 21, with the method defined by Goetz, et al., was used to evaluate motor and non-motor disability.^13^

2- NMS scale was established and validated for the first time by Chaudhuri, et al. in 2007.^10^ The scale evaluates the severity and frequency of NMS occurring in PD in the last month, is relatively easy to apply, takes about 10-15 minutes to complete, and is applied by the physician. The scale contains 30 questions divided into nine domains: cardiovascular (2 items), sleep/fatigue (4 items), mood/cognition (6 items), perceptual problem/hallucinations (3 items), attention/memory (3 items), gastrointestinal tract (3 items), urinary tract (3 items), sexual function (2 items), and miscellany (4 items).^10^

3- NMSQ, comprising a series of 30 questions, which is a screening tool for evaluating NMS, and is not used as a rating scale.^9^

4- PDQ-39 is used as a reliable and valid tool for the assessment of QOL in PD patients, with 8 discrete scales: mobility (10 items), activities of daily living (ADL) (6 items), emotional well-being (6 items), stigma (4 items), social support (3 items), cognitions (4 items), communication (3 items), and bodily discomfort (3 items).

Patients are asked to select one of 5 responses on a scale (never, occasionally, sometimes, often, always) for each event.^11^

The 5-MMSE questionnaire contains 11 questions that measure five aspects of cognitive function: orientation, registration, attention and calculation, recall, and language. The maximum score is 30, takes only 5-10 minutes to administer, and is consequently practical to use in clinics as a routine tool. The Persian version of the MMSE was used in this study, which has been validated for our society, based on age and education.^12^^,^^13^

All data were analyzed using the SPSS software (version 20, IBM Corporation, Armonk, NY, USA). Quantitative demographic characteristics were expressed by mean ± standard deviation (SD), and qualitative data were shown as percentage. To compare means of two normally distributed data, Student’s t-test was used, and for non-normally distributed data, the Mann-Whitney test and U-test were used. For comparisons of correlations between two groups, chi-square and Fisher’s exact tests were used, and Spearman’s rank correlation coefficient was applied to evaluate the associations among variables.

The total scores of UPRDS (I through V, I plus II, and total), PDQ39 (total number and each section), NMSS (total number and each domain), NMSQ total numbers, and total MMSE were calculated by summating items. A P value of < 0.05 was considered to be statistically significant.

## Results

***Patient characteristics*** *:* Eighty-one (n = 81) Isfahanian patients with PD, including 60 men and 21 women, with mean age of 62 ± 12 years (range 36 to 83 years) and mean disease duration of 6.1 ± 5.0 years (range 3 months to 20 years), enrolled in this study. Table 1 summarizes their demographic data and clinical characteristics. Disease severity evaluated by Hoehn and Yahr staging^7^ demonstrated that the highest proportion of patients were in stage 2 (n = 32, 39.5%), while the lowest proportions were in stages 4 and 5, with 4 patients in each of those stages. The mean score of UPDRS was 57.2 ± 26.0. The mean scores of motor symptoms and NMS of patients were 37.2 ± 20.0 and 20.1 ± 10.0, respectively. 

The mean MMSE score was 25 ± 5 (range 17-30). The most prevalent MMS score was 30 (18.7%), and there was not any correlation between disease duration and cognitive impairment (P = 0.607).

***NMS:*** All 81 patients (100%) had at least one non-motor symptom based on NMSS. The mean total score of the NMSS was 37.03 ± 22.51 with a range between 1 and 96. Among the domains of NMS, the highest percentages were seen in the domains of sleep/fatigue (87.7%). The lowest percentages were those from perception/hallucinations domain (34.6%). These results are demonstrated in table 2. The most frequently (> 60%) reported symptoms were fatigue (74.1%), constipation (67.9%), anxiety (65.4%), and short term memory loss (60.5%). Gastrointestinal tract and sexual function were significantly more prevalent in men (74% and 75%, respectively) than in women (26% and 25%, respectively) (chi-square test, P < 0.020). There were no differences between genders for the remaining NMS domains. Detailed frequencies of NMS are shown in table 3. 

**Table 1:** Characteristics of patients with Parkinson’s disease (PD)

**Characteristics**	**Value**
Age (year) (mean ± SD)	62 ± 12
Men (%)	74.1
Education [n (%)]	
Illiterate	24 (29.6)
Primary or/and secondary school	31 (38.3)
High school	16 (19.8)
University graduated	10 (12.3)
Duration of disease (year) (mean ± SD)	6.1 ± 5.0
Smoking (%)	23.5
Comorbidity [n (%)]	
Hypertension	11 (13.6)
Diabetes	9 (11.1)
Ischemic heart disease	5 (6.1)
Hyperlipidemia	6 (7.4)
Hoehn and Yahr stage (%)	
1	29.6
2	39.5
3	14.8
4	4.9
5	4.9
MMSE (mean ± SD)	25 ± 5
MDS-UPDRS (mean ± SD)	57.2 ± 26.0
Part I	9.5 ± 5.0
Part II	10.7 ± 7.0
Part III	35.0 ± 18.0
Part IV	2.3 ± 3.2
Non-motor symptoms total	20.1 ± 10.0
Motor symptoms total	37.2 ± 19.0
Antiparkinsonian medication (%)	
Levodopa	31.3
Dopamine agonist	57.5
Amantadine	54.3
Anticholinergic	52.6
MAO-B inhibitor	32.8

MMSE: Mini-Mental Status Examination; MDS-UPDRS: Movement Disorder Society-Unified Parkinson’s Disease Scale; SD: Standard deviation; MAO: Monoamine oxidase

***NMSQ:*** The most prevalent “yes” answer in the NMSQ was constipation (71.6%). The rarest symptom was loss of taste/smell (16.7%). Detailed data are provided in table 3.

Between the duration of disease and NMS there was a direct relationship (part I and II UPDRS P < 0.001, NMSS total P < 0.001, NMS-Q total P < 0.018).

***NMS and QOL (PDQ-39):*** The mean PDQ-39 score was 29.51 ± 18.51 and the median was 25. The most prevalent symptom was feeling pain in the body (77.6%) in the body discomfort domain, which was categorized in mobility domain. Also, there was an association between QOL and the duration of disease, so that as the disease progressed, the QOL worsened. Detailed data are shown in table 4. 

**Table 2:** Frequency of non-motor symptoms (NMS) by domains

**Domain**	**Percentage**
Cardiovascular	40.4
Lightheadedness/dizziness during the postural changes	35.8
Fall because of syncope	32.1
Sleep/fatigue	87.7
RLS	54.3
Insomnia	58.0
Excessive day time sleepiness	54.3
Fatigue	74.1
Mood/cognition	84.0
Anhedonia	38.3
Loss of motivation	48.1
Anxiety	65.4
Sadness/depression	58.0
Flat mood	46.9
Lack pleasure	39.5
Perceptual problem/hallucination	34.6
See something others can not	23.5
Believe you are not true	19.8
Double vision	18.5
Attention/memory	71.6
Difficulties to maintain concentration	53.1
Short-term memory problems	60.5
Forget to do daily things	46.9
Gastrointestinal tract	79.0
Drooling of saliva	54.3
Difficulty in swallowing	28.4
Constipation	67.9
Urinary tract	70.4
Urgency	50.6
Frequency (voiding every 2 hours)	48.1
Nocturia	49.4
Sexual function	44.4
Decreased pleasure	40.7
Problem having sex	44.4
Miscellaneous	69.1
Pain	34.6
Smell or taste dysfunction	17.3
Weight change	43.2
Excessive sweating	34.6

NMS: Non-motor symptoms; RLS: Restless leg syndrome

***Correlation between QOL and NMS: ***In this study several questionnaires for assessment of NMS were used, such as UPDRS parts I and II, NMSS, and NMSQ. We assessed correlation between NMS (by this form) and QOL (by PDQ-39), and the results showed strong correlations between them. The P-value for correlation between all NMS questionnaires and QOL was P < 0.001. Detailed results are revealed in table 5.

**Table 3:** Frequency of non-motor symptoms in patients with Parkinson’s disease (PD) non-motor symptoms questionnaire (NMSQ)

**Variable**	**Percentage**
Loss of taste/smell	16.7
Difficulty in swallowing	25.7
Vomiting/nausea	22.2
Constipation	71.6
Fecal incontinence	22.4
Incomplete bowel emptying	28.6
Urinary urgency	55.3
Nocturia	58.1
Unexplained pain	55.3
Change in weight	42.7
Memory	55.7
Apathy	38.6
Hallucination	21.1
Problems of concentration	41.6
Sadness	63.4
Anxiety	65.6
Change in libido	42.6
Sexual difficulties	43.1
Dizziness	38.0
Falls	45.1
Daytime sleepiness	44.6
Insomnia	50.7
Vivid dreams	40.8
Sleep behavior disorders	47.4
Restless legs	35.6
Edema	28.0
Excessive sweating	38.6
Diplopia	18.9

***Correlation between QOL and motor symptoms:*** We used Hoehn and Yahr staging,^7^ UPDRS III for evaluating motor symptoms, and UPDRS IV for motor complications and found correlations between Hoehn and Yahr staging, UPDRS III, UPDRS IV, and UPDRS total and PDQ-39 scale (P < 0.001, P < 0.001, P = 0.210, and P < 0.001, respectively). 

Hoehn and Yahr^7^ and UPDRS part III had the highest correlation in mobility and ADL (P < 0.001).

***Correlation between NMS and motor symptoms: ***We found correlations between NMSQ and Hoehn and Yahr^7^ (P = 0.390), UPDRS III (P = 0.008), and UPDRS total score (P = 0.001). Also, NMSS total correlated with UPDRS total, and UPDRS part III had a correlation only with domain 4 of NMSS total (P < 0.001).

**Table 4:** Impact of non-motor symptom in quality of life Parkinson’s disease questionnaire-39 (PDQ-39)

**Dimensions**	**Mean ± SD**	**Most finding**	**Percentage**
Mobility	13.5 ± 10.41	Difficulty looking after things	72.7
ADL	7.02 ± 5.78	Difficulty writing clearly	73.9
Emotional well-being	7.83 ± 5.54	Feeling anxious	73.3
Stigma	5.34 ± 6.10	Conceal his/her PD from the others	48.2
Social support	2.22 ± 3.02	Lacked support from his/her partner	41.2
Cognitions	4.67 ± 3.66	Memory deterioration	68.8
Communication	2.60 ± 2.75	Difficulty speech	64.9
Bodily discomfort	4.62 ± 3.13	Feeling pain in the body	77.6

SD: Standard deviation; PD: Parkinson’s disease; ADL: Activities of daily living

***Correlation between NMS and cognition: ***Out of 81 patients, 43 had no cognitive impairment (cut-off point 27), 15 of them had minimal cognitive impairment (cut-off point 24), and the remainder had MMSE 19-24. In this study we found correlations between the NMSQ total score, the PDQ-39 total score, and the MMSE, with P-values of 0.011 and 0.015, respectively. However, there was no significant relation between NMSS total score and MMSE (P = 0.175).

**Table 5:** Spearman’s rank correlation coefficient (rs) and P-value between non-motor symptoms scale (NMSS) domains and Parkinson’s disease questionnaire-39 (PDQ-39)

**NMSS domain**	**rs**	**P**
Cardiovascular	0.057	0.307
Sleep/fatigue	0.363	0.001
Mood/cognition	0.377	0.001
Perceptual problem/hallucination	0.392	< 0.001
Attention/Memory	0.395	< 0.001
Gastrointestinal tract	0.110	0.163
Urinary tract	0.158	0.080
Sexual function	0.120	0.148
Miscellaneous	0.116	0.150
NMSS total	0.468	< 0.001

NMSS: Non-motor symptoms scale

***Correlation between motor symptoms and cognition:*** Data analysis revealed correlations between motor symptoms and MMSE scores, as the Hoehn and Yahr^7^ scores were significantly higher in PD patients with lower MMSE scores (P = 0.004). Also, there was a significant association between UPDRS part III and MMSE scores (P = 0.003).

## Discussion

Recently, much has been written about NMS as disabling symptoms of PD that may affect QOL more than motor symptoms.

In about 20% of patients with PD, NMS may be the main presenting features.^14^ However, PD is usually diagnosed when motor symptoms appear, which is the time that most dopaminergic neurons are lost, but prior to this time, NMS would not be usually attended by clinicians.

Unfortunately, the situation is worse in developing countries. Most patients seek treatment after they become disabled from their motor symptoms, and NMS impact their QOL but they and their physicians do not pay attention to them.

To the best of our knowledge, this study is the first one on prevalence of NMS in Isfahan. We found high prevalence of NMS in our PD population as 100% of them had at least one NMS, with the most prevalent one being fatigue (87.7%). Most of them had been disabled by their untreated NMS.

Other studies showed the same result: Barone, et al. evaluated 1072 patients and found that nearly all of those patients complained of at least one NMS, where fatigue (58.1%) was the most prevalent one.^15^ Li, et al. in China reported the prevalence of NMS 100% in their PD sample, and again, fatigue (76.0%) was the most common NMS.^6^

Another study in Malaysia reported 97.3% NMS prevalence in their PD samples, where gastrointestinal symptoms were more prevalent (76.1%), and among them, constipation was the most common.^16^

In Peru, another study was done where NMSQ was used as the sole assessment tool for NMS, and they reported that 99.3% of their patients suffered from NMS, with depression and sadness being the most common symptoms.^17^ Estrada-Bellmann, et al., with the same methodology, showed that fatigue was the most common domain of NMS symptoms in a Mexican sample.^18^ Most of these studies showed the same results, but some differences may have occurred because of methodological differences between those studies and this research. Such differences include inclusion and exclusion criteria, using different questionnaires, racial variably, healthcare facilities, and economic conditions.

As shown in table 2, NMS symptoms had a high prevalence in our sample compared to previous studies. This indicates a lack of sufficient tertiary healthcare in our country, and unfamiliarity of our population with PD. Only when the disease imposes high impacts on their QOL and ADL they seek treatment. 

One study in Tehran, Iran, evaluated the QOL of patients with PD and showed that motor symptoms affecting activities of daily life, depression, anxiety, and being woman had impact on the QOL of PD patients, but NMSS and NMSQ were not used in that study and they mostly paid attention to the QOL of patients.

Fatigue is often recognized by patients with PD as one of their most disabling symptoms with the greatest impression on their QOL.^19^

Prevalence of fatigue in PD was reported to be between 33%–78%,^19^ but our results showed a higher prevalence (87.7%). Fatigue causes severe disability, although this symptom is one of the most prevalent NMS in PD, but has mostly been neglected by patients and clinicians. Kang, et al. were reported that fatigue can increase risk the risk of developing PD.^20^

The results have shown strong correlations between the duration of disease and NMS as well as PDQ-39, so, NMS begin before motor symptoms. NMS also develop throughout the course of disease and cause disability that adds on to motor symptoms. This is shown by the strong correlations between disease duration and the NMSS mood/cognition, perceptual problem/hallucination, attention/memory, and sexual domains, and similar domains in the NMSQ. Also, there were correlations between motor symptoms and NMS, which again indicate that as disability worsens (indicated by worse by motor symptoms), NMS become worse, too. As most PD drugs improve only motor symptoms, these findings could show why patients' QOL remain poor despite adequate treatment.

Cognitive impairment correlated with NMS and motor symptoms, but disease duration and MMSE score had no association, which may indicate that cognition correlates with disease severity. Thus, patients with more severe PD have worse NMS and motor symptoms, and less cognitive reserve.

We acknowledge that our study has some limits, including low sample size and lack of normal population as controls, however, this study is the first one in Iran that used the following questionnaires: NMSS, NMSQ, PDQ-39, and UPDRS I-IV for assessing NMS.

## Conclusion

In conclusion, as with other studies, we found a high prevalence of NMS in our sample. In our opinion, a change in PD criteria may be necessary, such as adding NMS to diagnostic criteria that could help to diagnose PD earlier, and when neuroprotection becomes available, diagnosis in earlier stages may help prevent worsening motor symptoms and disability. Also, faster and more accurate diagnosis and treatment of NMS would improve patients' QOL and prevent disability.

## Notes:

**How to cite this article:** Salari M, Chitsaz A, Etemadifar M, Najafi MR, Mirmosayyeb O, Bemanalizadeh M, et al. Evaluation of non-motor symptoms and their impact on quality of life in patients with Parkinson’s disease, Isfahan, Iran. Iran J Neurol 2017; 16(3): 118-24.
